# An Atypical Case of Pancreatic Cancer with Mesenchymal Differentiation in a Patient with Primary Lung Adenocarcinoma: Insights into Tumor Biology and Novel Therapeutic Pathways

**DOI:** 10.3390/diagnostics14222512

**Published:** 2024-11-09

**Authors:** Noura Abbas, Lama Zahreddine, Ayman Tawil, Mustafa Natout, Ali Shamseddine

**Affiliations:** 1 Department of Internal Medicine, Naef K. Basile Cancer Institute, American University of Beirut Medical Center, Riad El Solh, Beirut 1107-2020, Lebanon; na331@aub.edu.lb (N.A.); lz08@aub.edu.lb (L.Z.); 2 Department of Pathology and Laboratory Medicine, American University of Beirut Medical Center, Riad El Solh, Beirut 1107-2020, Lebanon; at04@aub.edu.lb; 3 Department of Radiology, American University of Beirut Medical Center, Riad El Solh, Beirut 1107-2020, Lebanon; mn113@aub.edu.lb

**Keywords:** pancreatic cancer, vimentin, mesenchymal, spindle cells, epithelial–mesenchymal transition (EMT), histopathology, diagnosis, treatment, case report

## Abstract

**Background**: Pancreatic cancer is among the malignancies with the poorest prognosis, largely due to its aggressive nature and resistance to conventional therapies. **Case Summary**: This report describes the case of a 69-year-old male patient with stage IV primary lung adenocarcinoma presenting with high levels of programmed death-ligand 1 (PD-L1). Simultaneously, abdominal computed tomography (CT) showed a dilated pancreatic duct at the level of the pancreatic head and a hypodense lesion in the uncinate process involving the superior mesenteric artery. Fine-needle aspiration (FNA) of the pancreatic lesions was negative. After three cycles of chemoimmunotherapy, positron emission tomography–computed tomography (PET-CT) showed complete remission of the lung nodules, lymphadenopathy, and pleural thickening, as well as a decrease in the size of the pancreatic lesion. After another six months, a PET-CT scan showed a focal increased uptake in the pancreatic mass in the same location, indicating disease progression. A core biopsy of the pancreatic tumor showed atypical spindle cell morphology with positive staining for vimentin, characteristic of mesenchymal differentiation with no apparent epithelial features. Comprehensive molecular profiling through Caris Molecular Intelligence^®^ revealed four genes with actionable mutations in the pancreatic tissue, including *KRAS* (p.G12D) and *TP53* (p.R175H). These molecular findings suggested the diagnoses of sarcomatoid carcinoma and conventional pancreatic ductal adenocarcinoma with epithelial–mesenchymal transition. Primary mesenchymal tumors and neuroendocrine neoplasms were excluded because immunohistochemistry was negative for anaplastic lymphoma kinase (ALK), smooth muscle actin (SMA), desmin, CD34, signal transducer and activator of transcription 6 (STAT6), S100, HMB45, CD117, discovered on GIST-1 (DOG1), CD56, progesterone, and synaptophysin. However, despite multiple rounds of systemic chemotherapy, immunotherapy, and radiation, his pancreatic disease rapidly deteriorated and metastasized to the liver and bone. **Conclusions**: Despite multiple lines of treatment, the patient’s condition worsened and he succumbed to his pancreatic malignancy. This study highlights the clinical characteristics, diagnosis, and treatment of rare pancreatic cancer, emphasizing the importance of molecular testing and histopathological biomarkers in personalizing treatment. It also provides insights into promising therapeutic approaches for similar cases with an unusual presentation.

## 1. Introduction

Pancreatic cancer remains a highly fatal disease, ranking among the malignancies with the worst outcomes [1]. The incidence of pancreatic cancer increases with age, with a median age of 70 years, and it is more common in men than in women. The incidence and mortality rates are alarmingly close, largely because approximately 80% of patients present with unresectable or metastatic disease [1,2], associated with a 5-year survival rate of less than 5% [3]. This malignancy is also characterized by its insidious onset, aggressive nature, and resistance to conventional therapies [1].

The most common type of pancreatic cancer is pancreatic ductal adenocarcinoma (PDAC), which accounts for over 90% of all cases. PDAC is characterized by glandular structures and desmoplastic stroma, contributing to its aggressive behavior and poor prognosis. Neuroendocrine neoplasms, which make up less than 5% of pancreatic cancers, have a more indolent course but can vary widely in their behavior and prognosis depending on their grade and stage. Acinar cell carcinoma, another rare type, tends to have a better prognosis than PDAC but can still be aggressive. Mesenchymal tumors, including sarcomas, are extremely rare and have distinct pathological features and clinical outcomes, often depending on the specific type of mesenchymal tissue involved [4].

The main diagnostic methods for suspected pancreatic cancer include imaging tests such as computed tomography (CT) scanning and magnetic resonance imaging (MRI), endoscopic ultrasound (EUS), biopsy procedures, and blood tests for tumor markers like CA19-9. However, these methods have limitations. CT scans and MRIs may not detect small lesions or differentiate between benign and malignant tumors effectively. EUS, while more sensitive, is invasive and operator-dependent. Biopsies can be challenging due to the tumor’s location and may not always provide a definitive diagnosis. Tumor markers like CA19-9 lack specificity and can be elevated in other conditions, leading to false positives [1]. These limitations underscore the need for new diagnostic approaches to improve early detection and accuracy.

A key biological process implicated in the dismal prognosis of PDAC is the epithelial-to-mesenchymal transition (EMT), where cells acquire a mesenchymal phenotype and migratory properties. This process, along with several markers and prognostic factors, contributes to the enhanced invasiveness and metastatic potential of pancreatic tumors. EMT is described as downregulation in epithelial markers with increased expression of mesenchymal markers such as vimentin. This process is one of the triggers of early progression and metastasis in PDAC [5].

In advanced stages, systemic chemotherapy is the mainstay of treatment when curative resection is no longer possible. However, the response to treatment is highly heterogeneous, and some cases may exhibit atypical features, increasing the challenges in diagnosis and treatment [6]. This variability can be attributed to genetic and molecular differences, the presence of various subtypes of pancreatic cancer, and the tumor microenvironment. Emerging therapies, including targeted treatments and immunotherapies, are being investigated to address these challenges, but their efficacy varies widely among patients [1]. Recent studies have shown promise for combination therapies that target multiple pathways involved in PDAC progression. Understanding molecular and genetic variations is crucial for developing personalized treatment approaches and improving patient outcomes [1,6].

## 2. Case Presentation

In this context, we present the case of a 69-year-old former heavy-smoking man, with a medical history of hypertension, diabetes, dyslipidemia, an overweight status (BMI 26.5 kg/m^2^), and coronary artery disease managed with percutaneous transluminal coronary angioplasty. The patient had no notable family history of malignancy.

In December 2016, he presented with persistent flank pain and a troublesome productive cough that had been present for six months, along with a recent episode of hemoptysis. A CT scan of the chest, abdomen, and pelvis revealed a dilated pancreatic duct at the level of the pancreatic head and a 2 × 1.6 cm hypodense lesion in the uncinate process involving the superior mesenteric artery over less than half its circumference. Additionally, multiple pulmonary nodules were identified, including a 2 × 2 × 3.7 cm irregular subpleural mass in the right lower lobe with associated pleural thickening and a 1.3 × 1.3 cm pleural-based solid nodule in the right upper lobe with an adjacent right hilar lymph node. These findings were confirmed by a positron emission tomography–computed tomography (PET-CT) scan, which showed focal nodular activity in the uncinate process (SUV max = 3.7) and a posterior pleural-based lesion in the right lower lobe (SUV max = 10) with large ipsilateral hilar active adenopathy, but no evidence of retroperitoneal adenopathy, metastatic liver, or skeletal disease.

Subsequent fine-needle aspiration (FNA) of the pancreatic lesion yielded only macrophages and benign surface epithelial cells, likely of duodenal origin. A CT-guided biopsy of the pulmonary lesion revealed programmed death-ligand 1 (PD-L1)-positive lung adenocarcinoma, with more than 50% of tumor cells expressing PD-L1 (Supplementary Figure S1). The tumor stained positive for thyroid transcription factor 1 (TTF1), cytokeratin 7 (CK7), and Napsin A, but negative for CDX-2, cytokeratin 20, and anaplastic lymphoma kinase (ALK), and presented a wild-type epidermal growth factor receptor (EGFR). Given the ipsilateral hilar adenopathy and pleural-based metastasis, the lesion was classified as T4N1M1a (stage IVA). Laboratory workup showed normal tumor markers, including CA19-9 at 20.9 IU/mL (N < 37), carcinoembryonic antigen (CEA) at 2.4 ng/mL (N < 7 for smokers), and lactate dehydrogenase (LDH) at 146 IU/L (N = 110–265).

Upon diagnosis, the patient started systemic treatment with cisplatin 145 mg, pemetrexed 900 mg, and pembrolizumab 200 mg, as per the KEYNOTE-189 trial protocol [7], from January to February 2017. After three cycles, a PET-CT scan indicated complete remission of the lung nodules, lymphadenopathy, and pleural thickening, as well as a decrease in the size of the pancreatic lesion, with faint to minimal uptake. This positive initial response prompted the initiation of maintenance therapy with pembrolizumab 200 mg from March to December 2017.

**Figure 1:**
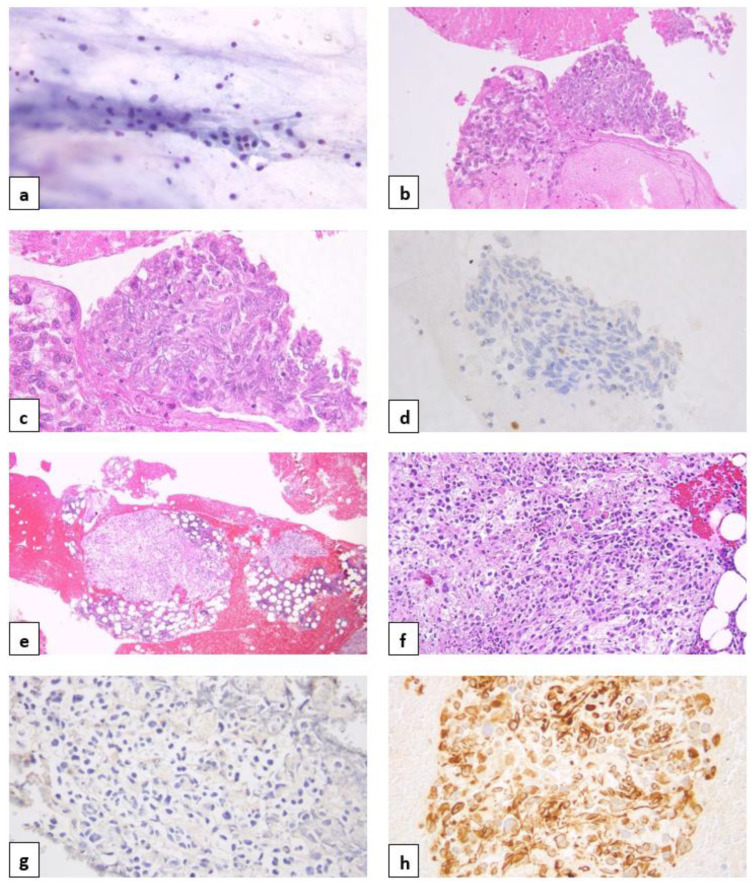
Histopathological analysis of the second pancreatic fine-needle aspiration (FNA) and the left iliac bone core biopsy. (**a**) The FNA smear from the pancreatic mass showing clusters of atypical spindle cells; (**b**) the hematoxylin and eosin (H&E) section of the pancreatic cell block (low magnification), (**c**) the H&E section of the pancreatic cell block (high magnification), (**d**) negative cytokeratin AE1/AE3 staining in the pancreas, (**e**) the H&E section of the left iliac bone (low magnification), (**f**) the H&E section of the left iliac bone (high magnification), (**g**) negative cytokeratin AE1/AE3 staining in the bone, and (**h**) positive vimentin staining in the bone.

In September 2017, a PET-CT scan showed a focal increased uptake in the pancreatic mass in the same location, indicating disease progression. These findings were confirmed in the CT scan of October 2017, which further demonstrated the recurrent mass in the pancreas, now measuring 3.7 × 4.1 × 3.8 cm, involving the pancreatic head and uncinate process, and abutting the superior mesenteric artery for approximately 180 degrees, with a few adjacent sub-centimetric peripancreatic lymph nodes. A core biopsy of the pancreas identified an atypical spindle cell tumor (Figure 1), clinically staged as T4N1M0 (stage III).

The pancreatic FNA specimen exhibited distinctive features, including positivity for vimentin and CD10, and negativity for cytokeratin AE1/AE3, cytokeratin 8/18, desmin, synaptophysin, TTF1, and PD-L1. This staining profile was completely different from the primary lung cancer (Table 1). The tumor also showed mesenchymal differentiation and atypical traits such as nuclear pleomorphism, visible mitotic activity, and focal necrosis. The patient presented elevated amylase and lipase levels, with values of 290 IU/L (N = 10–120) and 308 IU/L (N = 13–60), respectively.

**Figure 2:**
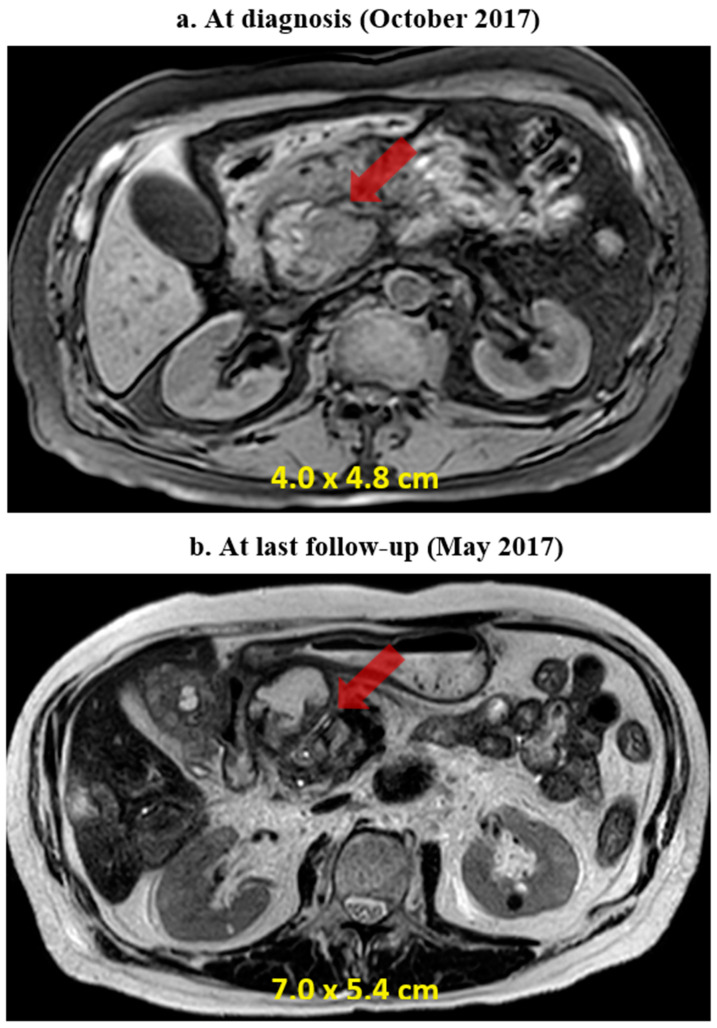
Comparative magnetic resonance imaging (MRI) of the abdomen (**a**) at diagnosis (October 2017) and (**b**) at the last follow-up (May 2018), showing disease progression despite several lines of treatment. The arrow indicates the pancreatic tumor.

Two weeks following the initial assessment, MRI of the abdomen revealed a 4 × 4.8 cm mass in the pancreatic head (Figure 2), with several sub-centimetric peripancreatic and retroperitoneal lymph nodes, and extensive inflammatory changes with central necrosis indicative of acute necrotizing pancreatitis. The inflammation affected the peripancreatic tissue while sparing the pancreatic parenchyma. After recovering from this acute episode, the patient underwent stereotactic body radiation therapy (SBRT) targeting the pancreatic mass, receiving a total dose of 30 Grays in five fractions in November 2017.

Concurrently, comprehensive tumor profiling with Caris Molecular Intelligence^®^ was performed on the second pancreatic FNA tissue sample, analyzing DNA, RNA, and proteins across a 592-gene panel via next-generation sequencing and immunohistochemistry. This revealed actionable mutations in four genes, *CIC* (exon 15, c.3467-1G>A), *KRAS* (exon 2, p.G12D), *RB1* (exon 24, c.2490-1G>A), and *TP53* (exon 5, p.R175H), as well as variants of unknown significance in 18 additional genes (Supplementary Table S1). The tumor was characterized as microsatellite-stable and had an intermediate tumor mutational burden of 11 mutations per megabase.

Despite treatment efforts, the patient’s clinical condition worsened. In January 2018, MRI of the abdomen showed new metastatic liver lesions, with the largest measuring 1.4 × 1.7 cm. Given the patient’s initial positive response to cisplatin and pemetrexed, and considering the potential benefit of these therapies as suggested in the next-generation sequencing (NGS) profile (Supplementary Table S2), the patient was again started on a regimen of cisplatin 135 mg, pemetrexed 900 mg, and pembrolizumab 900 mg, which he received for two cycles. However, due to a lack of response and progression of the metastatic disease, the treatment was switched to nanoparticle albumin-bound paclitaxel (nab-paclitaxel) 230 mg and gemcitabine 1800 mg for seven cycles [8], alongside pembrolizumab maintenance for three cycles. A subsequent PET-CT scan in May 2018 demonstrated an increase in both the number and size of liver lesions, with progression of the pancreatic head lesion to 5.9 × 5.1 cm and new bone metastases. The pancreatic and liver lesions exhibited multiple areas of necrosis (with low uptake) and blood. MRI of the pelvis also disclosed multiple enhancing bone lesions affecting the pelvic region, lower lumbar spine, and proximal femur, with the largest seen in the posterior aspect of the left iliac bone measuring 2 cm, indicative of metastatic spread.

A CT-guided core biopsy with bone marrow aspirates from the left iliac bone lesion confirmed the pancreatic origin of the metastatic cells, which were positive for vimentin and CD10 and negative for cytokeratin AE1/AE3 and TTF1 (Table 1).

By the end of May 2018, the patient presented with fatigue, reduced oral intake, and intermittent watery diarrhea, which were managed symptomatically. Further MRI of the abdomen revealed continued growth of the pancreatic mass, now measuring 7 × 5.4 cm, numerous abdominal and retroperitoneal lymph nodes, and the development of additional metastatic lesions in the spine, iliac bones, and liver. The patient was hospitalized for persistent abdominal and bone pain.

Unfortunately, in June 2018, the patient passed away due to the progression of his disease.

## 3. Discussion

Pancreatic cancer, particularly PDAC, is known for its aggressive nature and resistance to therapy [1]. The complexity of this disease is further highlighted by the current case, presenting challenges in diagnosis and treatment. An overview of the treatment received and disease evolution is illustrated in Supplementary Figure S1.

The reduction in both lung and pancreatic lesions in the PET-CT scan of March 2017, following treatment with cisplatin, pemetrexed, and pembrolizumab, initially suggested primary lung cancer with metastasis in the pancreas, which led to the decision to maintain the patient on this treatment. Metastasis from the lungs to the pancreas is a rare situation (<5% of pancreatic tumors), more commonly reported in small-cell lung cancer than in lung adenocarcinoma, and is often accompanied by metastasis at other sites [9]. Although some differences in biomarkers between primary and metastatic non-small-cell lung cancer have been described [10], the patient’s subsequent clinical progression, the distinct histological morphology, and the absence of TTF1 in the pancreatic tumor tissue suggest a diagnosis of primary pancreatic neoplasm, leading to a change in treatment to gemcitabine and nab-paclitaxel. Moreover, the substantial difference in the SUV max value between the lung and pancreatic lesions (10 vs. 3.7, respectively) on the initial PET-CT scan supports the existence of two separate primary tumors. The FNA of the pancreas conducted in December 2016 yielded negative results. However, the target was likely missed given that the recurrence occurred at the same location as the previously identified pancreatic lesion. The reliance on PET-CT can pose limitations in accurately evaluating pancreatic cancer due to its lower sensitivity for detecting small lesions and its inability to provide detailed anatomical information [11].

The differential diagnosis of pancreatic cancer in this case was complicated. The tumor presented as atypical spindle cells expressing vimentin and CD10, but without expression of epithelial markers (cytokeratin AE1/AE3, cytokeratin 8/18, desmin). This pattern could suggest either a primary mesenchymal tumor or a transition of cells from an epithelial to mesenchymal phenotype. Mesenchymal tumors of the pancreas are very rare, representing less than 1% of all pancreatic neoplasms. They are classified according to their biomarkers, histological morphology, and cytological features.

Following the histology findings, several diagnoses could be ruled out, including inflammatory myofibroblastic tumors, leiomyosarcoma, tumors of perivascular epithelioid cells (due to negative expression of ALK, smooth muscle actin (SMA), desmin, and HMB45), solitary fibrous tumors, gastrointestinal stromal tumors (negative CD34, CD117, and discovered on GIST-1 (DOG1)), and schwannoma (negative S100) [12]. Among mesenchymal tumors of the pancreas, a potential diagnosis is undifferentiated spindle cell sarcoma, which is less likely given the presence of a *KRAS* mutation, which is uncommon in pancreatic sarcoma. The option of a neuroendocrine tumor was also ruled out given the negativity of CD56, synaptophysin, and progesterone (Table 1).

Conventional PDAC, an infiltrating tumor of epithelial origin that forms glandular, duct-like structures [13], remains the most common type of pancreatic cancer. The spindle cell morphology observed in this patient’s biopsy is not typical of PDAC, but it could be partially explained by EMT. The mutation patterns in *KRAS* and *TP53*, which are typically seen in pancreatic carcinomas and rarely in sarcomas, further support this diagnosis. However, the lack of nuclear staining for beta-catenin is not characteristic of EMT. In normal cases, beta-catenin is present in the cell membranes and not in the nucleus. When EMT occurs, there is an activation of the Wnt signaling pathway that leads to the translocation of beta-catenin into the cytoplasm and its accumulation in the nucleus [14].

A rare and highly malignant variant of PDAC is sarcomatoid carcinoma, which is a poorly differentiated tumor characterized by extensive loss of glandular differentiation, nuclear pleomorphisms with a high mitotic rate, and the presence of atypical spindle-shaped tumor cells [13,15]. These tumors are known to be very aggressive, with several cases expressing positivity for CD10. There is also a recurrent presence of *KRAS* and *TP53* mutations in sarcomatoid carcinoma, similar to conventional PDAC [15]. Although this tumor usually exhibits both epithelial and mesenchymal components, some cases with negative or less pronounced epithelial expression have been reported [15,16]. In our case, there were only mesenchymal features, a situation further complicated by the paucity of tissue samples and the unresectable status of the tumor, which can hamper accurate diagnosis.

Skeletal metastases are relatively rare in pancreatic cancer, occurring in approximately 5% of cases. They typically manifest in the late stages of the disease, with a median survival time of 4.6 months [17]. In this case, the patient survived for only one month after the development of metastases in the pelvis and spine. Additionally, the change in treatment from cisplatin and pemetrexed to gemcitabine and nab-paclitaxel in February 2018 led to the emergence of areas of blood and necrosis in the pancreatic mass and liver metastases. This response, particularly the increase in blood within the mass following treatment, is not commonly reported in the literature.

The patient’s prognosis was influenced by several factors, including the positivity for mesenchymal staining and CD10 [18], the mutation in *TP53* [19], the high neutrophil–lymphocyte ratio at diagnosis and throughout the disease course (≥5) [20], and the unresectable status with positive lymphadenopathy at diagnosis. These factors can enhance the progression of pancreatic cancer and resistance to treatment. Despite receiving several therapies with potential benefit (Supplementary Table S2) and radiation therapy (SBRT), the patient’s condition continued to worsen.

As we move forward, new potential treatments may offer hope to patients with unresectable pancreatic cancer. Although immunotherapy has an emerging role in various solid tumors, its application in pancreatic cancer remains controversial, particularly in microsatellite-stable tumors with negative PD-L1. Mutations in *KRAS* and *TP53*, reported in 90% and 50–70% of PDAC cases, respectively, have been the subject of several clinical trials as potential treatment targets (Supplementary Table S2). *KRAS* is frequently mutated in various cancers and is emerging as a target for cancer therapy. Two treatments targeting the *KRAS* G12C mutation have been approved for non-small-cell lung cancer, namely sotorasib (AMG510) and adagrasib (MRTX849) [21]. However, in pancreatic cancer, the most common *KRAS* mutation is G12D, accounting for 45% of all *KRAS* mutations. Recent advancements have led to the development of potential treatments for the *KRAS* G12D mutation, such as MRTX1133, TH-Z827, TH-Z835, and KD-8, which are currently under preliminary experiments and have shown promise in preclinical models [22].

Although EMT and mesenchymal markers in pancreatic cancer have been shown to correlate with poor prognosis and clinical outcomes, the translation of these findings into clinical practice is still under investigation [23].

The abovementioned histopathological findings made PDAC with EMT a reasonable diagnosis. Both the pancreatic FNA and the left iliac bone lesion biopsy specimens exhibited mesenchymal differentiation and were positive for vimentin, a mesenchymal/EMT marker, without displaying typical epithelial characteristics.

Although several case reports have documented the involvement of the EMT process in pancreatic cancer [24,25,26,27,28,29] (Supplementary Table S3), cases that precisely identify instances of EMT in conventional PDAC have been sparsely reported in the literature.

Given the overexpression of vimentin, coupled with the patient’s deteriorating condition despite multiple lines of treatment and the urgent need for novel therapeutic strategies, we conducted a retrospective study aiming to determine the expression levels of EMT markers as predictors of survival outcomes in patients with resectable PDAC from January 2005 to June 2019. The findings indicated a strong correlation of vimentin overexpression and EMT phenomenon with reduced overall and disease-free survival in this patient group [30].

Furthermore, we are currently preparing a prospective study to further validate the prognostic value of vimentin in a population with resectable PDAC. This study will explore the prognostic impact of EMT, its correlation with molecular subtypes, and the potential for personalized therapeutic approaches. 

## 4. Conclusions

In conclusion, this case underscores the aggressive and complex nature of pancreatic cancer, which often resists conventional therapies. It highlights the importance of considering rare diagnoses when confronted with atypical presentations, the value of a comprehensive and multidisciplinary approach to patient care, and the need for continued research into new therapeutic strategies. This case also emphasizes the potential applicability of molecular markers in informing clinical decisions, providing valuable insights for future therapeutic strategies, and improving patient outcomes.

## Figures and Tables

**Table 1:** Histological markers presented by lung, pancreas, and left iliac bone tumors.

Lung	Pancreas	Left Iliac Bone
TTF1 +	TTF1 -	TTF1 -
CK7 +		CK7 -
Napsin A +		Napsin A -
PD-L1 > 50%	PD-L1 -	
	Vimentin + ^m^	Vimentin + ^m^
	CD10 + ^p^	CD10 + ^p^
	Cytokeratin AE1/AE3 - ^e^	Cytokeratin AE1/AE3 - ^e^
	Cytokeratin 8/18 - ^e^	
	Desmin - ^e, t^	
	Beta-catenin: Membrane positivity, no nuclear staining	
ALK -	ALK - ^t^	
CDX-2 -		
CK20 -		
EGFR wild type		
	CD4 -	
	CD23 -	
	CD30 -	
	CD34 - ^t^	
	CD56 - ^n^	
	CD68 -	
	CD117 - ^t^	
	Progesterone - ^n^	
	S100 - ^t^	
	SMA - ^t^	
	Synaptophysin - ^n^	
		STAT6 - ^t^
		HMB45 - ^t^
		DOG1 - ^t^

+, positive markers; - negative markers; ALK, anaplastic lymphoma kinase; DOG1, discovered on GIST-1; e, epithelial marker; m, mesenchymal marker; n, neuroendocrine marker; p, prognostic marker; SMA, smooth muscle actin; STAT6, signal transducer and activator of transcription 6; t, tumor type marker for mesenchymal tumors of the pancreas (detailed here). Inflammatory myofibroblastic tumor (IMT): ALK +, SMA +, and desmin +; solitary fibrous tumor (SFT): CD34 + and STAT6 +; schwannoma: S100 +; leiomyosarcoma: SMA + and desmin +; tumor of perivascular epithelioid cells (PEComa): SMA + and HMB45 + (HMB45 is also a marker of melanoma); gastrointestinal stromal tumor (GIST): CD34 +, CD117 +, and DOG1 +.

## Data Availability

The data supporting the findings of this study are available upon request from the corresponding author.

## Supplementary Materials

The following supporting information can be downloaded at: https://www.mdpi.com/article/10.3390/diagnostics14222512/s1, Figure S1: Overview of treatment received and disease evolution. Table S1: The patient’s gene mutations in the Caris Molecular Intelligence^®^ profile. Table S2: Therapies with potential benefit tailored to the patient’s molecular profile according to the Caris Molecular Intelligence^®^ tumor report. Table S3: Summary of case reports documenting epithelial–mesenchymal transition in pancreatic cancer.
