# Supplementary material for: An Atypical Case of Pancreatic Cancer with Mesenchymal Differentiation in a Patient with Primary Lung Adenocarcinoma: Insights into Tumor Biology and Novel Therapeutic Pathways
Source: Diagnostics (Basel). 2024 Nov 9;14(22):2512. doi: 10.3390/diagnostics14222512 (PMC11592984; doi:10.3390/diagnostics14222512)
Supplement: Supplementary file 1 [file diagnostics-14-02512-s001.zip › diagnostics-3225384-supplementary.pdf]

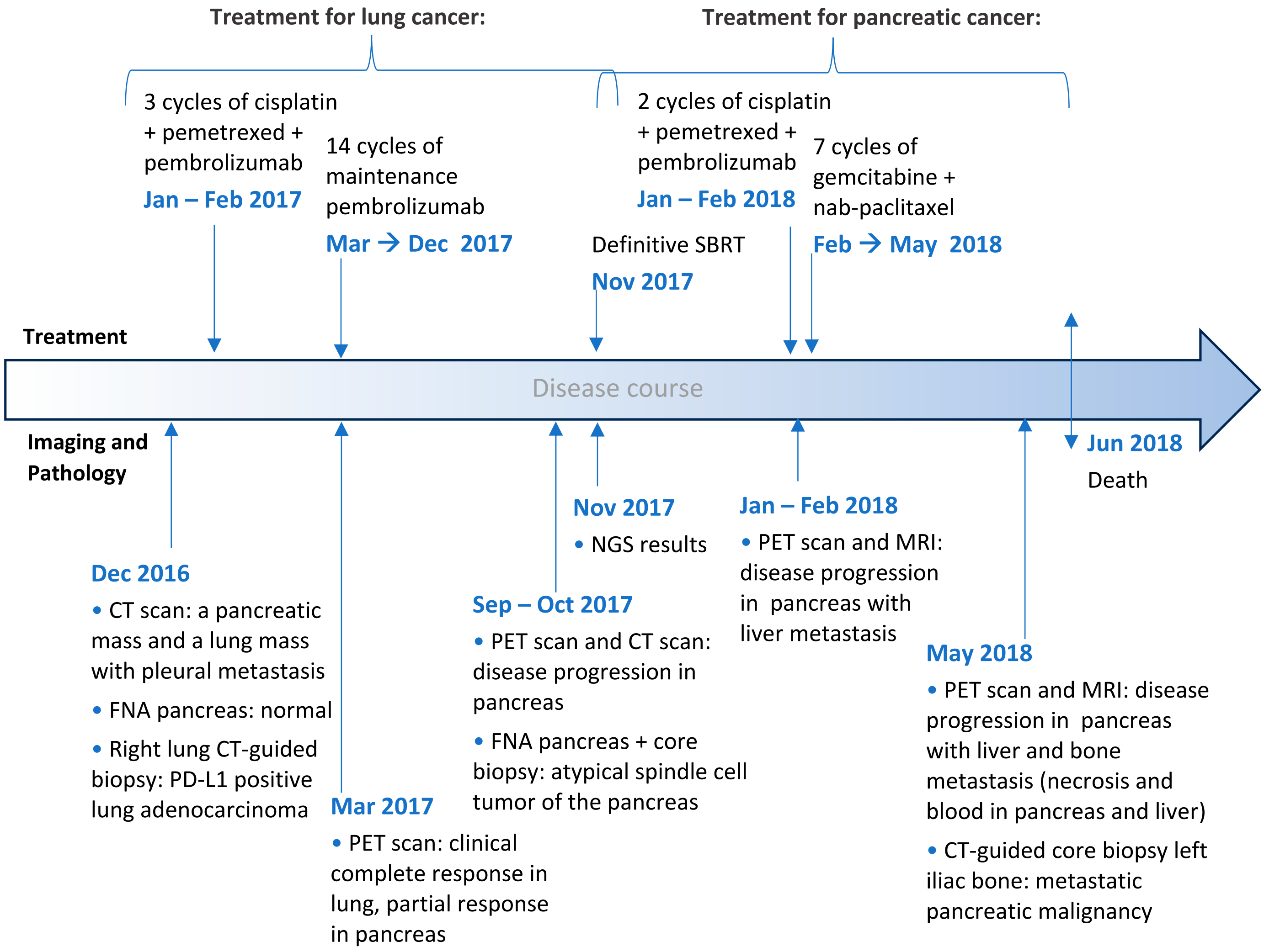

Supplementary Figure S1. Overview of treatment received and disease evolution.

CT, computed tomography; FNA, fine-needle aspiration; NGS, next-generation sequencing; PD-L1, programmed-death ligand 1; PET, positron emission tomography.

Supplementary Table S1. The patient's gene mutations in the Caris Molecular Intelligence® profile.

Gene name	Amino acid change	Base change	Exon	VAF	Pathogenicity
CIC	–	c.3467-1G>A	15	52%	Pathogenic
KRAS	p.G12D	c.35G>A	2	32%	Pathogenic
RB1	–	c.2490-1G>A	24	44%	Pathogenic
TP53	p.R175H	c.524G>A	5	28%	Pathogenic
ABL2	p.V150M	c.493G>A	–	–	VUS
ARHGAP26	p.D784N	c.2185G>A	–	–	VUS
ATRX	p.D332G	–	–	–	VUS
AURKA	p.G173R	–	–	–	VUS
BARD1	p.R656H	–	–	–	VUS
BCL9	p.P1135	–	–	–	VUS
BCR	p.I413M	c.1239C>G	–	–	VUS
CNTRL	p.R1863Q	c.5588G>A	–	–	VUS
CREBBP	p.A1907T	c.5719G>A	–	–	VUS
	p.A63G	–	–	–	VUS
DOT1L	p.Q629E	–	–	–	VUS
IGF1R	p.Q576L	–	–	–	VUS
MECOM	p.K877N	–	–	–	VUS
	p.N724K	–	–	–	VUS
NIN	p.R100S	c.300A>T	–	–	VUS
PCM1	p.N1975K	–	–	–	VUS
PDE4DIP	p.D1842V	–	–	–	VUS
PTCH1	p.P1384L	c.4151C>T	–	–	VUS
RNF213	p.R2034Q	–	–	–	VUS
SMARCA4	p.G1612S	c.4834G>A	–	–	VUS

– data not available; VAF, variant allele frequency; VUS, variant of unknown significance.
No mutation was detected in 557 genes, particularly in: ATM, BRAF, BRCA1, BRCA2, c-KIT, cMET, EGFR, HER2/Neu (ERBB2), IDH1, NRAS, PDGFRA, PIK3CA, and RET.

Supplementary Table S2. Therapies with potential benefit tailored to the patient's molecular profile according to the Caris Molecular Intelligence® tumor report.

Biomarker	Result	Drug class	Investigational agents
ERCC1	IHC: negative (0+, 100%)	Platinum compounds	Carboplatin, **cisplatin**, oxaliplatin
TUBB3	IHC: negative (2+, 10%)	Taxanes	Docetaxel, **paclitaxel**
TS	IHC: negative (1+, 3%)	Antifolates	Methotrexate, **pemetrexed**
		Pyrimidine analog	Capecitabine, fluorouracil
RRM1	IHC: negative (0+, 100%)	Nucleoside analog	**Gemcitabine**
MGMT	IHC: negative (0+, 100%)	Alkylating agents	Dacarbazine, temozolomide
KRAS	NGS: pathogenic mutation	ERK inhibitors	BVD-523
		MEK inhibitors	GDC-0973, PD0325901, XL518, selumetinib, trametinib
		Multikinase inhibitors	Regorafenib
TP53	NGS: pathogenic mutation	Cell cycle inhibitors	LY2606368, MK-1775
		p53-targeted biological agents	Ad5CMV-p53, modified vaccinia virus Ankara vaccine expressing p53

( ), represents the highest staining intensity with the corresponding percentage of cells; IHC, immunohistochemistry; NGS, next-generation sequencing.

The treatments highlighted in bold are the ones received by this patient.

Supplementary Table S3. Summary of case reports documenting epithelial-mesenchymal transition in pancreatic cancer.

Study	Tumor type and stage	Tumor markers	Treatment	Response to treatment
Ren et al., 2013 [1]	Sarcomatoid carcinoma, early-stage	AACT+, pan-CK+, CK19+, CK8/18+, and vimentin+, CD68–, and lysozyme–	- Complete resection of the tumor<br>- Adjuvant gemcitabine, oxaliplatin, and floxuridine	Good response to treatment (DFS >3 years)
Sicklick et al., 2013 [2]	PDAC, stage II at diagnosis	Not reported	- Gemcitabine	Disease progression
			- Notch inhibitor	Transient partial response
Lu et al., 2014 [3]	Adenosquamous carcinoma with sarcomatoid change	Sarcomatoid lesions: CK7+, CA19-9–, E-cadherin–, vimentin+	- Distal pancreatectomy<br>- Traditional medicine and thymosin	Disease progression and liver metastasis after 5 months
Naito et al., 2018 [4]	Pancreatic anaplastic carcinoma, stage IV at diagnosis	Sarcomatoid lesions: vimentin+, ZEB1+, E-cadherin–	Not reported	OS 3 months
Kimura et al., 2020 [5]	Sarcomatoid carcinoma, stage II at diagnosis	Sarcomatoid lesions: CKAE1/AE3+, vimentin+, PD-L1–	- Distal pancreatectomy<br>- Adjuvant gemcitabine	Good response to treatment (DFS >10 years)
Kimura et al., 2021 [6]	Sarcomatoid carcinoma, early-stage	Sarcomatoid lesions: pSmad2/3+, Snail+, fibronectin+, γ-H2AX+, p53+, p21+	- Distal pancreatectomy<br>- Adjuvant gemcitabine	Good response to treatment (DFS >11 years)
	Sarcomatoid carcinoma, locally advanced stage	Sarcomatoid lesions: pSmad2/3+, Snail+, fibronectin+, γ-H2AX–, p53–, p21–	- Neoadjuvant TS-1<br>- Distal pancreatectomy<br>- Adjuvant chemotherapy with hepatic arterial infusion	Disease progression and liver metastasis, OS 18 months
	Sarcomatoid carcinoma, unresectable	Sarcomatoid lesions: pSmad2/3+, Snail+, fibronectin+, γ-H2AX–, p53–, p21–	- Intraperitoneal injection of cisplatin	Disease progression and liver metastasis, OS 2 months

AACT, α-1-antichymotrypsin; CK, cytokeratin; DFS, disease-free survival; OS, overall survival; PDAC, pancreatic ductal adenocarcinoma; PD-L1, programmed cell death ligand 1; TS-1, tegafur, gimeracil, and oteracil.

References

1. Ren CL, Jin P, Han CX, Xiao Q, Wang DR, Shi L, et al. Unusual early-stage pancreatic sarcomatoid carcinoma. World J Gastroenterol. 2013 Nov 21;19(43):7820–4.

2. Sicklick JK. Correcting the misnomers of epithelial-mesenchymal relations. J Surg Res. 2013 Jun 1;182(1):36–9.

3. Lu BC, Wang C, Yu JH, Shen ZH, Yang JH. A huge adenosquamous carcinoma of the pancreas with sarcomatoid change: An unusual case report. World J Gastroenterol. 2014 Nov 21;20(43):16381–6.

4. Naito Y, Kawahara A, Taira T, Takase Y, Murata K, Ishida Y, et al. Cytopathological and immunocytochemical findings of pancreatic anaplastic carcinoma with ZEB1 expression by means of touch imprint cytology. Diagn Cytopathol. 2018 Feb;46(2):198–203.

5. Kimura T, Fujimoto D, Togawa T, Ishida M, Iida A, Sato Y, et al. Sarcomatoid carcinoma of the pancreas with rare long-term survival: a case report. World J Surg Oncol. 2020 May 25;18(1):105.

6. Kimura T, Togawa T, Iida A, Noriki S, Sato Y, Goi T. Does cellular senescence play an important role in the prognosis of sarcomatoid carcinoma of the pancreas? World J Surg Oncol. 2021 Mar 16;19(1):79.
